# Abdominal tuberculosis manifested as tuberculosis of the urachal sinus in an adolescent and the role of laparoscopy in the management: a rare case report

**DOI:** 10.1186/s12879-016-1405-6

**Published:** 2016-02-05

**Authors:** Sze Li Siow, Hon Leong Sha, Chee Ming Wong

**Affiliations:** 1 Department of Surgery, Jalan Hospital, Kuching, 93586 Sarawak Malaysia; 2 Department of Surgery, Faculty of Medicine and Health Sciences, Universiti Malaysia Sarawak, Kota Samarahan, Kuching, 94300 Sarawak Malaysia

**Keywords:** Tuberculosis, Urachal sinus, Urachal abscess, Infection, Laparoscopy

## Abstract

**Background:**

Abdominal tuberculosis (TB) is an uncommon affliction in adolescence. It is usually associated with pulmonary tuberculosis. The disease is caused by lymphohaematogenous spread after primary infection in the lung or ingestion of infected sputum and has a typically protean and nonspecific presentation. The occurrence of TB in an urachal remnant is probably from the contiguous spread of an abdominal focus or mesenteric lymph node. Urachal TB is a rare entity, with only two reported cases in the literature. We report here a case of clinically silent pulmonary and abdominal TB that manifested in the infection of an urachal sinus and highlight the role of laparoscopy in its diagnosis and treatment.

**Case presentation:**

A 14-year-old boy presented to our institution with peri-umbilical swelling and purulent discharge from his umbilicus for 2 weeks duration. There were no radiological, microbiological or clinical evidences of TB in the initial presentation, though he had close social contact with someone who had TB. A computed tomography scan of the abdomen confirmed the diagnosis of an urachal abscess. An incision and drainage procedure was performed followed by a course of antibiotics. A scheduled laparoscopic approach later showed that the peritoneum and serosal surface of the small and large intestines were studded with nodules of variable sizes, in addition to the urachal sinus. The histology of the resected tissues (urachal sinus and nodules) was consistent of TB infection. He recovered fully after completing 6 months of anti-tuberculous therapy.

**Conclusion:**

This report highlights a rare case of TB urachal abscess in an adolescent boy, the difficulties in the diagnosis of abdominal tuberculosis, the need to consider TB as a cause of urachal infection in endemic areas and the use of laparoscopy in both diagnosis and treatment.

## Background

Tuberculosis (TB) remains a major burden to healthcare in affected countries with an estimate of 9.0 million cases and 1.5 million deaths globally in 2013 [1]. Most cases were in South-East Asia and Western Pacific Regions (56 %), where Malaysia is located [1]. The estimated incidence in Malaysia was 29 thousand cases or 99 cases per 100,000 populations in 2013 [2]. The annual incidence of new TB cases in Malaysia is increasing since 2005 [2, 3], with a similar finding observed in the paediatric group [3]. Close contact with a person with active TB, social factors and male sex are important risk factors for TB infection in children and adolescents [3, 4]. While pulmonary TB and lymph nodal TB are the commonest presentations in children [3], abdominal TB is an uncommon presentation that complicates pulmonary TB in 1–5 % of cases [5, 6]. Even rarer is the association of TB and the urachus, with only two cases reported in the English language literature [7, 8] (Table 1). We report the case of an adolescent boy with a TB urachal abscess in the setting of abdominal TB and highlight the role of laparoscopy for diagnosis of abdominal TB and treatment of the urachal remnant.

**Table 1:** Comparison of case reports of urachal tuberculosis

Author, publication year	Presentation	Age, gender	Imaging modality	Surgical management	Mode of diagnosis of TB	Outcome
Fujimoto et al., [7] 2000	Pollakiuria and non-tender infra-umbilical abdominal mass	62-year-old, Male	MRI of the abdomen	Open En bloc resection with partial cystectomy	PCR test	Recovered well
Jindal et al., [8] 2013	Intermittent peri-umbilical pain, low grade fever, and loss of weight	23-year-old, Male	CT of the abdomen	Open En bloc resection	Histological examination characteristic of TB	Recovered well
Present case	Peri-umbilical pain, swelling and purulent discharge from umbilicus	14-year-old, Male	CT of the abdomen	Laparoscopic resection of urachal cyst, and biopsy of the nodules over the peritoneum and falciform ligament	Histological examination characteristic of TB	Recovered well

*TB* tuberculosis, *MRI* magnetic resonance imaging, *CT* computed tomography, *PCR* polymerase chain reaction

## Case presentation

A 14-year-old Malaysian boy with no known medical history presented with fever, peri-umbilical discharge, pain and swelling for 2 weeks. There was no history of chronic cough, night sweats, weight loss or symptoms of urinary tract infection. He was the second of 5 siblings. The family stayed in a crowded long-house with access to piped water. The family had history of contact with a neighbour who had pulmonary TB. However, his parents and siblings did not show any ill effects from the exposure. Clinical examination showed an adolescent boy of medium build. There was no generalised lymphadenopathy. The abdomen was soft with a palpable ill-defined tender swelling deep to the umbilicus. There was an opening at the base of the umbilicus with purulent discharge.

**Fig. 1:**
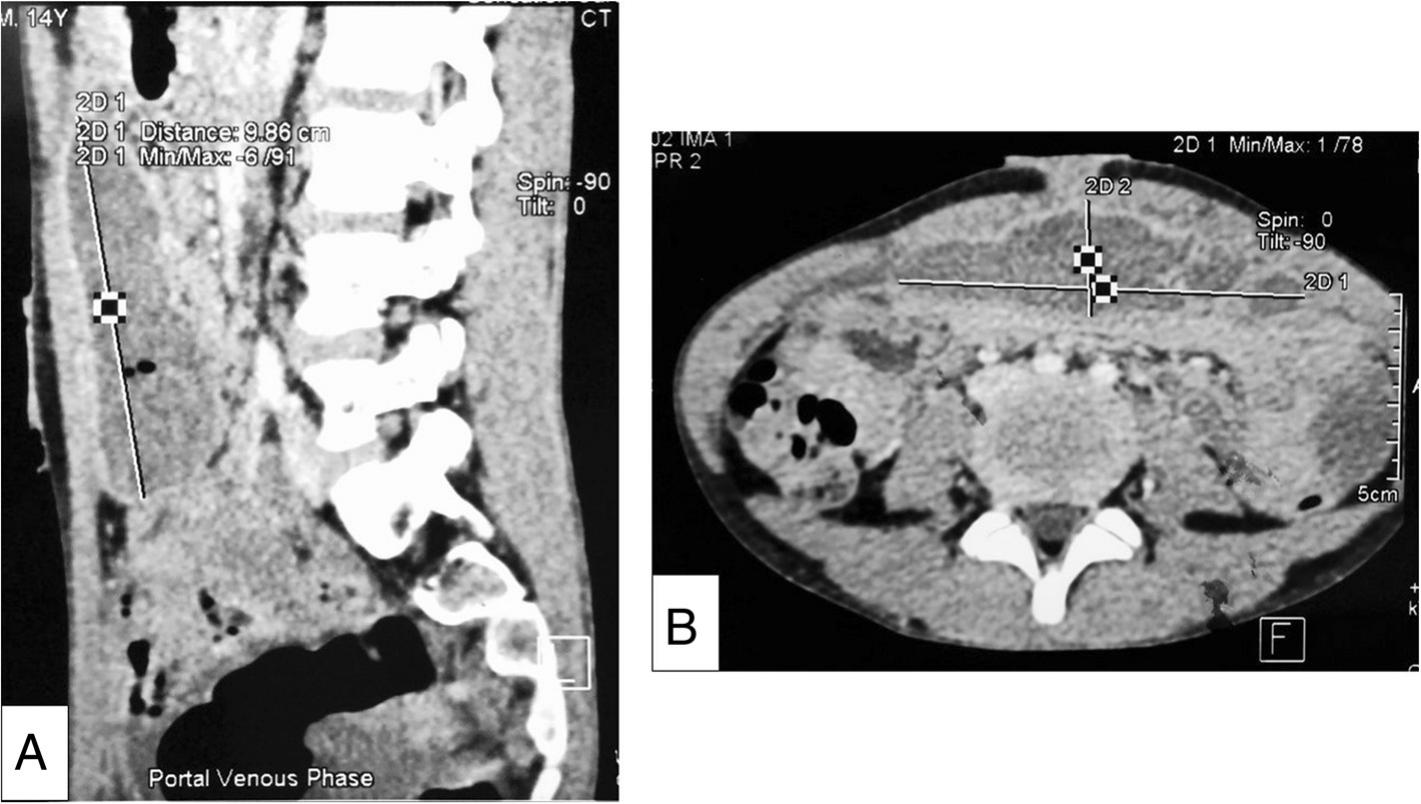
Computed tomography scan of abdomen showing the urachal abscess. **a** Sagittal view **b** Transverse view

Chest radiograph did not demonstrate features suggestive of pulmonary TB. Computed tomography scan (CT) of the abdomen revealed an urachal sinus measuring 10.8 × 3.1 × 9.9 cm with a collection containing pockets of air (Fig. 1). The initial impression was that of an urachal abscess, and the boy underwent incision and drainage, followed by a course of oral antibiotics. Acid fast bacilli were not detected in smear examination of the pus and sputum samples. Standard bacterial cultures of the pus isolated from the umbilicus showed no growth after 5 days of incubation. Tuberculin skin test was negative. Screening for Human Immunodeficiency Virus was negative. He recovered well and was allowed home after 7 days of hospitalization with a plan for delayed excision of the urachal sinus once the infection has resolved.

**Fig. 2:**
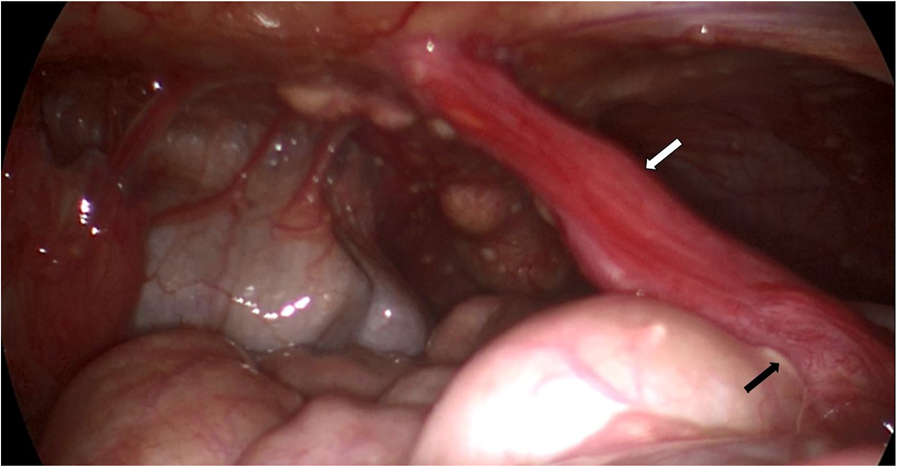
Laparoscopic view showing the parietal and visceral peritoneum studded with tubercles. Urachal sinus (white arrow) was seen attached to the anterior surface of the sigmoid colon (black arrow)

Two months later, he underwent an elective laparoscopy with the intention of excising the urachal sinus. Intra-operative laparoscopy revealed multiple sub-centimetre nodules on the peritoneum and serosal surfaces of the small and large bowels. The urachal sinus was noted to be partly detached from the abdominal wall with its inferior end adherent to the sigmoid colon (Fig. 2). No ascites was noted. Complete excision of the urachal sinus was performed using an ultrasonic dissector. Multiple biopsies of the nodules over the peritoneum and falciform ligament were taken using a combination of sharp dissection and energy devices.

The post-operative recovery was uneventful. Histopathological examination of the urachal sinus, peritoneal nodules and falciform ligament showed multiple epithelioid granulomas with central caseous necrosis and Langhans giant cells . The Ziehl-Neelsen stain performed on these granulomas was positive for acid-fast bacilli (AFB). The sputum culture was subsequently positive for AFB after 4 weeks of incubation.

The diagnosis of concurrent active pulmonary and abdominal TB was made. The patient was then started on a 6-month course of anti-tuberculous therapy (Pyrazinamide 800 mg OD, Ethambutol 800 mg OD, Rifampicin 300 mg OD and Isoniazid 150 mg OD) after consultation with an infectious disease consultant. During the follow-up examination 6 months later, there were no discharge from the umbilicus and he had recovered completely.

## Discussion

Abdominal TB is defined as TB infection involving the gastrointestinal tract, peritoneum, mesentery, abdominal lymph nodes, and solid visceral organs such as liver, pancreas and spleen [9]. It is sixth most common type of extrapulmonary TB, affecting primarily young adults [5, 9]. It is relatively rare in children [5, 9]. While the ileocecal junction is the most common site reported for abdominal TB [10, 11], the peritoneum and lymph nodes are the most common sites involved in children with abdominal TB [9, 12]. Mycobacterium tuberculosis and bovis (transmitted through unpasteurized dairy products) are the main pathogens involved. The diagnosis is often delayed because of its non-specific and protean clinical presentation [6]. It is a condition that mimics a variety of inflammatory, infectious and neoplastic gastrointestinal diseases [9, 13]. Fever, abdominal pain and weight loss are the most common symptoms found in children with abdominal TB [6, 12]. There are three patterns of clinical presentation depending on the predominant symptoms: intestinal (colicky abdominal pain, vomiting and gaseous abdominal distension), peritoneal (abdominal distension and ascites) and asymptomatic [13]. Our patient was virtually asymptomatic and had no apparent radiological features of pulmonary TB nor clinical evidences of abdominal TB, other than the urachal discharge. The diagnosis was established during laparoscopy performed for excision of the urachal remnant.

Delay in diagnosis of abdominal TB is associated with high morbidity and mortality, if left untreated [5, 14, 15]. Diagnosis is often made difficult because the signs and symptoms are non-specific and often resemble those of other gastrointestinal diseases. It is even more difficult in children because of the paucibacillary nature of the disease and lower culture yields. Most children have constitutional symptoms such as fever, anorexia and weight loss (60–70 %) [11], while some have no obvious symptoms or risk factors [12]. There are no reliable tools for the diagnosis of abdominal TB. Tuberculin skin positivity varies from 42–88 % in different studies [11, 13, 16] and has a lower specificity for abdominal disease compared to pulmonary TB [16]. Chest radiograph is a routine and simple imaging performed but only 15–56 % of patients with abdominal TB are reported to have chest radiographic evidence of pulmonary TB [5, 9, 16]. Ultrasound and CT imaging are important ancillary tools in the diagnosis. CT is the most common imaging used in most studies [6, 16]. The CT features of ascites, lymphadenopathy, bowel wall thickening, or omental and mesenteric stranding raise suspicion of abdominal TB [6, 16]. However, such findings often resemble those of bacterial peritonitis or advanced malignant disease [5, 9, 14]. Therefore, the diagnosis of abdominal TB is often established after a correlation of clinical features, laboratory data and imaging findings. In the era of laparoscopy, diagnostic laparoscopy has emerged as an important diagnostic armamentarium and should be considered when the radiological findings are ambiguous. It allows obtaining tissue specimen for histological confirmation and ascitic fluid for acid-fast smears and cultures [13]. It is a procedure with low morbidity and a high diagnostic yield of 70 to 95 % [10]. Peritoneal biopsy by laparoscopy has a higher diagnostic yield of 85–100 % [15] compared to 3–20 % [13] in acid-fast smears and cultures of ascitic fluid. The use of adenosine deaminase assay in ascitic fluid improves the diagnostic yield [15] but it is expensive and not readily available. Polymerase chain reaction (PCR) testing of the biopsied tissue is another useful tool with high specificity and sensitivity [11]. The laparoscopic approach in our case proved to be an ideal surgical approach as it also allows treatment of the urachal remnant other than taking targeted biopsies of peritoneal nodules under direct vision. Thus, sampling errors are minimized to facilitate the diagnosis of abdominal tuberculosis in an unsuspected patient. In addition, laparoscopic peritoneal biopsy results in a more rapid diagnosis of abdominal TB as compared to conventional microbiological assays which may take up to 4–6 weeks. More importantly, it avoids the morbidity and mortality associated with conventional laparotomy, in particularly potential wound-related complications.

The association of abdominal TB and urachal remnants is rare. The pathogenesis of abdominal tuberculosis in our patient was presumably via lymphohaematogenous spread from a primary focus in the lung or ingestion of infected sputum. He most probably had active pulmonary TB which was not radiologically apparent that subsequently spread to the abdomen. Both the infections had remained clinically silent until the abdominal TB manifested in the infection of urachal remnant. TB of the urachus probably resulted from contiguous spread from an abdominal focus or mesenteric lymph node.

The urachus, a remnant of the allantois, functions to excrete urine from the bladder via the umbilicus during the intra-uterine life of a foetus. After birth, the allantois may fail to involute and depending on the completeness of this involution, a patent urachus, urachal cyst, urachal sinus or vesico-urachal diverticulum may arise. Occasionally, these structures can be infected, and prompt the diagnosis and surgical intervention.

Infection is the most common complication of urachal remnants. It is the usual mode of presentation in an otherwise asymptomatic condition. Staphylococcus aureus is the most common organism cultured, though other organisms have been reported. The association of a urachal remnant and M. tuberculosis is a rare occurrence, with only two documented case reports in a PubMed search of the English literature [7, 8].

Successful treatment of infected urachal remnants involves an initial incision and drainage of the abscess followed by antibiotics with surgical resection best performed after the resolution of infection and inflammation [17]. Performing a definitive surgery in the acute setting is associated with technical difficulties and risk of injury to adjacent visceral organs, particularly the bladder [17]. Laparotomy has been the conventional method of treatment but laparoscopic approach offers advantages of minimally invasive technique and should be preferred whenever such expertise is available.

## Conclusion

We present a rare case of association of abdominal TB and urachal remnant. It highlights the difficulties of recognizing abdominal TB despite the fact that an initial CT scan was done for urachal abscess. The patient had a negative Mantoux test and lacked significant abdominal symptoms and signs suggestive of TB. M. tuberculosis was not identified from the initial sputum smear even though it was cultured later. Laparoscopy is a good therapeutic modality for urachal remnants and a useful diagnostic procedure in ambiguous cases of abdominal TB.

## Consent

Written informed consent was obtained from the patient’s parent for publication of this case report and accompanying images. A copy of the written consent is available for review by the Editor-in-Chief of this Journal.

## Abbreviations

AFB: acid-fast bacilli
CT: computed tomography
HIV: Human immunodeficiency virus
MRI: magnetic resonance imaging
PCR: polymerase chain reaction
TB: tuberculosis
